# Associations between State-Level Obesity Rates, Engagement with Food Brands on Social Media, and Hashtag Usage

**DOI:** 10.3390/ijerph182312785

**Published:** 2021-12-03

**Authors:** Yuanqi Gu, Jaime Coffino, Rebecca Boswell, Zora Hall, Marie A. Bragg

**Affiliations:** 1 Department of Population Health, New York University Grossman School of Medicine, New York, NY 10016, USA; yuanqi.gu.gk@gmail.com (Y.G.); Jaime.Coffino@nyulangone.org (J.C.); Rebecca.Boswell@pennmedicine.upenn.edu (R.B.); Zora.Hall@nyulangone.org (Z.H.); 2 School of Global Public Health, New York University, New York, NY 10003, USA

**Keywords:** obesity, social media

## Abstract

Food advertisement exposure is associated with increased caloric intake, but little is known about food/beverage placements in the digital media environment. We aimed to examine the correlation between the number of people who follow food and beverage brand social media accounts (i.e., user engagement) and state-level obesity rates; quantify social media followers’ use of “healthy” vs. “unhealthy” hashtags; and analyze the relationship between user engagement and hashtag usage. We identified the 26 fast-food and beverage brands with the highest advertising expenditures and used Demographics Pro to determine the characteristics of social media users amongst the 26 brands. A series of regression analyses were conducted that related the mean percentage of brand followers and state-level obesity rates. We then identified 733 hashtags on Instagram and 703 hashtags on Twitter, coding them as “healthy”, “unhealthy”, “neutral”, or “unrelated to health”. Intercoder reliability was established using ReCal2, which indicated a 90% agreement between coders. Finally, we conducted ANCOVA to examine the relationship between the mean percentage of brand followers and their hashtag usage. There was a significant, positive correlation between the state-level obesity rate and the mean percentage of followers of sugary drink or fast-food brands on Instagram and Twitter, but such a correlation between obesity and low-calorie drink brand followers was only found on Twitter. Our findings illustrate the relationship between the social media food environment and obesity rates in the United States. Given the high rates of engagement with food brands on social media, policies should limit digital advertisements featuring fast-food, sugary drink, and low-calorie drink brands.

## 1. Introduction

Obesity rates are high in the United States, with 42.4% [1] of adults and 18.5% [2] of children classified as having obesity (body mass index (BMI) > 30; Center for Disease Control and Prevention (CDC), 2020). Obesity is associated with an increased risk of negative health consequences [3,4] and higher medical care costs in the United States [5]. The risk factors for obesity are varied, but the food environment is one of the most notable, as it determines the accessibility and availability of both healthy and unhealthy food in a person’s diet.

The food environment is, in part, determined by proximity to fast-food restaurants and convenience stores, with shorter distances associated with an increased risk of obesity [6]. A number of studies demonstrated that a lower density of supermarkets increased the likelihood of an unhealthy diet for the residents of the given community [7,8,9]. Other studies have indicated that poor walkability of neighborhoods is a predictor of higher rates of obesity and diabetes [10,11,12]. Even the outdoor food advertising landscape has been named as a major public health concern for promoting unhealthy products in neighborhoods that already experience high rates of obesity. In one study that identified food advertisements in four cities, researchers found the density of advertisement was highest in African American and Latino zip code areas [13]. Thus, understanding and improving the food environment is of significant public health interest.

Previous research shows that social-media-based food advertising is growing. In 2007, food and beverage companies had just one social media account, but in 2016 they collectively maintained 568 accounts [14]. This dramatic rise in official corporate accounts led to significantly more people following food and beverage accounts, as indicated by a sample of 27 food and beverage brands that have an estimated 73.1 million followers [15]. In that study, researchers identified a positive correlation between youth-targeted advertising practices and the number of adolescent followers of those 27 brands on Twitter [15]. Our study is the first, however, to examine the correlation between state-level obesity rates and the number of people who follow food and beverage brands on social media.

Emerging research on social media engagement with food and beverage brands has shown how the unique features of different platforms—especially Instagram—enable advertisers to powerfully interact with users, affecting their brand awareness and ad preferences. Fleming and Harris (2020) conducted a cross-sectional survey, distributing it online among a diverse group of 1564 adolescents aged 13–17. The survey assessed how participants engage with restaurant, food, and beverage brands on social media. The results showed that 70% of the adolescent respondents reported engaging with brands. Further, the regression analyses revealed that among the survey participants, Black and “less-acculturated Hispanic” adolescents were more likely than White adolescents to engage with brands on social media [16]. In another online survey, Bragg et al. (2021) conducted an experiment testing whether Black and White adolescent respondents could distinguish between food and beverage brand images from traditional advertising mediums and Instagram. The findings suggested that among the 832 participants aged 13–17 years, they performed worse than chance when asked to identify whether a brand image originated from Instagram. When rating the images in the survey, though, adolescent respondents reported preferring images from Instagram over traditional mediums [17]. In separate analyses using the same sample of participants, adolescents viewed and rated food brand images from Instagram that either did or did not show user comments, as well as images that showed either high, medium, or low numbers of “likes.” The researchers found that adolescent respondents rated ads with medium or high numbers of “likes” higher than ads with few “likes.” In addition, the participants who reported heavy social media use were more than six times more willing to comment on brands’ Instagram posts compared to the participants who reported light social media use [18]. Finally, in a case study, researchers examined a week’s worth of user-generated Instagram content responding to one of KFC’s hashtag campaigns. Content analyses of 128 posts with #HowDoYouKFC revealed that 45% of them were “explicitly positive” toward the KFC brand and that 39% lacked a particular stance or emotional attitude toward KFC, showing that brands can turn individual Instagram users into positive advertisers of unhealthy and potentially harmful products [19].

Social Norms Theory can help explain why consumers—especially youth—are highly susceptible to the influence of social-media-based advertising. Social Norms Theory suggests that individuals tend to match or mimic behaviors perceived as the norm among their peers [20,21,22]. In social media ecosystems, the “like” button may capitalize on people’s sensitivity to peer behavior [23,24,25,26]. The interactivity of social media also perfectly lends itself to the adolescent desire for popularity—teens can comment on or like each other’s posts online [27]. Additionally, sometimes, when adults or adolescents comment on brands’ posts, they can receive momentary viral fame [28].

Despite robust evidence that the physical environment is associated with obesity rates, no studies have documented the association between obesity rates and engagement with food and beverage brands on Instagram and Twitter. The ubiquity of social media among adults and youth—coupled with the unprecedented rise in social-media-based food marketing—makes it imperative that public health experts document the extent of such marketing and begin to measure its possible effects on weight gain at the population level. This study aimed to take on this call by examining the relationship between state-level obesity rates and social media engagement with food and beverage brands. In this study, we also quantified the relationship between social media followers’ use of “healthy” vs. “unhealthy” hashtags, and whether that predicted their likelihood to follow sugar-sweetened beverage, fast-food, or low-calorie drink brands.

## 2. Methods

### 2.1. Data

We identified 27 fast-food, snack, and beverage brands with the highest advertising expenditures based on previous research [15]. Zero-calorie beverages and beverages with artificial sweeteners were classified as low-calorie drink brands; other beverages were classified as sugary drink brands. As there was only one snack brand, we excluded it from this study.

Demographic Pro provided characteristics of social media users (e.g., age, location) who followed the included 26 brands, including 23 brands on Instagram and 17 brands on Twitter (Table S1 in the supplemental materials). We downloaded the data in January 2019 and analyzed them from June to August 2020. Demographics Pro is a data analytics firm that uses a series of proprietary algorithms to estimate or infer the demographic characteristics of target audiences based on their social media presence/usage. The firm collects data signals from the following three primary areas: networks (the nature and the strength of ties between individuals), consumption (accounts followed and social media usage), and language (words used in posts and bios). Demographics Pro has used their methodology to profile over 300 million social media users. They require a confidence of 95% or above to make an estimate for each demographic characteristic of any single social media user. The success of their analytic predictions relies on the relatively low covariance of multiple amplified signals. Iterative evaluation using established samples allows the firm to balance depth of coverage (the number of demographic estimates made) and required accuracy. The sample size of social media users with verified demographic information ranges from 10,000 to 200,000 people, depending on the demographic characteristic under examination. Demographic Pro’s method performs equally well when evaluating the demographic characteristics of users on Twitter and Instagram.

In addition to demographic characteristics, we collected obesity rates by state from the Prevalence of Self-Reported Obesity by State and Territory, BRFSS, 2018 [29].

### 2.2. Hashtag Coding

We compiled a 1500-item list of our selected companies’ most frequently used hashtags, and then selected 773 Instagram and 703 Twitter hashtags that overlapped among all brands in the sample. We coded hashtags as “healthy”, “unhealthy”, “neutral”, or “unrelated to health.” All fruits and sports were coded as healthy, all meat as unhealthy, and all alcoholic drinks as neutral except for beer, which was coded as unhealthy. We tested intercoder reliability using ReCal2, an online reliability testing tool [30]. The results indicated a 90% agreement between the two participating coders. We found 79 healthy, 51 unhealthy, 49 neutral, and 593 unrelated-to-health hashtags on Instagram, and 57 healthy, 11 unhealthy, 9 neutral, and 626 unrelated-to-health hashtags on Twitter. The following analysis focuses solely on healthy and unhealthy hashtags, with neutral or unrelated hashtags omitted.

### 2.3. Statistical Analysis

We reported the number and percentage of followers by state in the U.S. on both Instagram and Twitter for each brand. We also reported the number and percentage of their followers who used a healthy or unhealthy hashtag. We computed brands’ followers/any users ratios by dividing the percentage of each brand’s followers by the mean percentage of users who followed any account on Instagram or Twitter. This allowed us to exclude the effects of hashtag popularity by evaluating the dominance of hashtags for each brand.

In the following statistical analyses, we employed the mean percent of followers of low-calorie drink, sugary drink, and fast-food brands on Instagram or Twitter. To examine the relationship between mean percentage of brand followers and state-level obesity rates, we first conducted a series of regression analyses. We found a highly positive correlation between the mean percentage of followers of sugary drink brands and fast-food brands. To avoid multicollinearity, we ran regression analyses with the mean percentage of followers of one of the three types of brands on Instagram or Twitter as predictors of obesity rates, with the mean percentage of users who followed any account as a control variable.

We conducted two-way analyses of covariance (ANCOVA) to examine whether the mean percentage of brand followers using a healthy or unhealthy hashtag was equal across all three types of brands. The independent variables were type of hashtags (healthy or unhealthy) and type of brands (low-calorie drink, sugary drink, or fast food). The covariant was the mean percentage of users who followed any account and used a healthy or unhealthy hashtag. The dependent variable was the mean percentage of followers of each kind of brand in our sample who used a healthy or unhealthy hashtag.

## 3. Results

### 3.1. Followers of Brands and Obesity Rate by State

Table 1 shows the mean number of brand followers in our sample by state—ranked by high to low obesity rates. It is important to note that the highly ranked states did not always have a higher mean percentage of brand followers, because the average number of social media users differed by each state. Having the most Instagram (18.9%) and Twitter (17.50%) users, California scores the highest mean number of followers of low-calorie drink (*n* = 4171, 12.83%), sugary drink (*n* = 202,417, 15.73%), and fast-food (*n* = 235,967, 3.09%) brands on Instagram, as well as low-calorie drink (*n* = 11,169, 12.48%) and sugary drink (*n* = 59,213, 12.04%) brands on Twitter. Texas has the highest mean number of followers for fast-food brands on Twitter (*n* = 124,858, 10.15%).

Through regression analyses, after controlling for the mean percentage of users who followed any account (Table S1 in the supplemental materials), we found a significantly positive correlation on Instagram between the mean percentage of followers of sugary drink (*p* = 0.01) or fast-food (*p* = 0.002) brands and state-level obesity rates. No such correlation was found for low-calorie drink brands (*p* = 0.22). On Twitter, the positive correlation was significant for all three brand categories (low-calorie drink: *p* = 0.009; sugary drink: *p* = 0.008; fast food: *p* = 0.003).

### 3.2. Followers of Brands and Their Hashtag Usage

Table 2 indicates the mean number of brand followers in our sample who used a healthy or unhealthy hashtag. Compared to the low-calorie drink and sugary drink brands, the fast-food brands had the largest total number of followers (Instagram: *n* = 30,145,454; Twitter: *n* = 9,031,753) and the greatest number of followers who used a healthy (Instagram: *n* = 278,839; Twitter: *n* = 11,500) or unhealthy (Instagram: *n* = 331,493; Twitter: *n* = 6982) hashtag. On Instagram, Starbucks had the largest total number of followers (*n* = 17,425,064) as well as followers using a healthy (*n* = 198,844) or unhealthy (*n* = 229,017) hashtag. On Twitter, Monster Energy had the highest total number of followers (*n* = 3,198,430) as well as followers who used a healthy (*n* = 4168) or unhealthy (*n* = 2337) hashtag.

**Figure 1:**
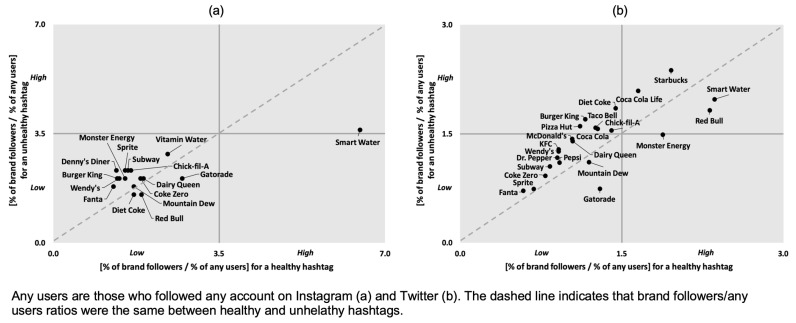
Relationship between the brand followers/any users ratio for a healthy hashtag and that for an unhealthy hashtag on Instagram (**a**) and Twitter (**b**).

Figure 1 shows the relationship (on Instagram and Twitter) between the brand followers/any users ratio for both healthy and unhealthy hashtags. On Instagram (Figure 1a), Smart Water (a low-calorie drink brand) and Gatorade, Monster Energy, Mountain Dew, and Red Bull (sugary drink brands) had more followers who used a healthy hashtag than those who used an unhealthy one. On Twitter (Figure 1b), Diet Coke and Smart Water (low-calorie drink brands), and Gatorade and Red Bull (sugary drink brands) had more followers who used a healthy hashtag than those who used an unhealthy one. The remaining brands showed the reverse relationship on social media: the number of followers who used an unhealthy hashtag was higher compared to those who used a healthy one.

**Figure 2:**
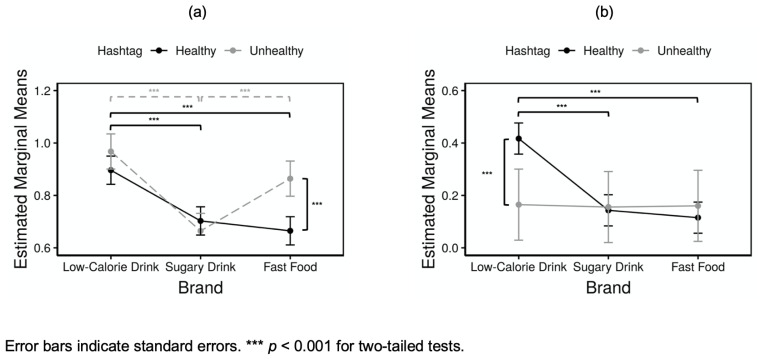
Estimated marginal means of each brand on Instagram (**a**) and Twitter (**b**).

The results of ANCOVA are represented in Figure 2 (Instagram and Twitter)(Table S2 in the supplemental materials). While controlling for the effects of the mean percentage of users who used any hashtag, we found a significant hashtag/brand interaction in terms of the mean percentage of brand followers using a healthy or unhealthy hashtag (*F* (2, 383) = 7.383, *p* < 0.001 for Instagram and *F* (2, 197) = 4.740, *p* = 0.01 for Twitter).

An analysis of the simple main effects for hashtags and brands was then conducted, with *p*-values corrected using a Bonferroni method (Tables S3 and S4 in the supplemental materials). On Instagram, the simple main effect of hashtags was significant in the fast-food brands (*F* (1, 127) = 32.2, *p* < 0.001), but not in the low-calorie drink brands (*F* (1, 127) = 3.83, *p* = 0.16) or the sugary drink brands (*F* (1, 127) = 0.853, *p* = 1.00). On Twitter, however, no significant effect was found in the low-calorie drink (*F* (1, 65) = 3.46, *p* = 0.20), sugary drink (*F* (1, 65) = 0.668, *p* = 1.00), or fast-food (*F* (1, 65) = 0.012, *p* = 1.00) brands. The simple main effect of brands was significant for the brand followers who used a healthy (*F* (2, 233) = 17.2, *p* < 0.001) or unhealthy (*F* (2, 149) = 32.9, *p* < 0.001) hashtag on Instagram. On Twitter, the effect was also significant for the brand followers who used a healthy hashtag (*F* (2, 167) = 26.2, *p* < 0.001), but not for those who used an unhealthy hashtag (*F* (2, 29) = 0.113, *p* = 1.00).

Pairwise comparisons were computed for significant simple main effects with Bonferroni-adjusted *p* values (Tables S5 and S6 in the supplemental materials). On Instagram, the findings revealed that the mean percentage of the fast-food brand followers who used a healthy hashtag was significantly lower than those who used an unhealthy one (*p* < 0.001) and, among those who used an unhealthy hashtag, the mean percentage of the followers of the sugary drink brands was significantly lower than that of the low-calorie drink (*p* < 0.001) or fast-food (*p* < 0.001) brands. On Twitter, the mean percentage of the low-calorie drink brand followers who used a healthy hashtag was significantly higher than those who used an unhealthy one (*p* < 0.001). On both Instagram and Twitter, among the users who used a healthy hashtag, there were significantly more followers of the low-calorie drink brands compared to followers of the sugary drink (*p* < 0.001) and fast-food brands (*p* < 0.001).

## 4. Discussion

This is the first study, to our knowledge, to examine the relationship between consumers’ engagement with food and beverage ads and obesity rates as well as their hashtag usage in the digital media environment. On Instagram, we identified a significant, positive correlation between the mean percentage of followers of sugary drink or fast-food brands and state-level obesity rates. There was no such correlation for low-calorie drink brands. On Twitter, the positive correlation between the states’ obesity rates and the number of followers was significant for low-calorie drinks, sugar-sweetened beverages, and fast food. In addition, we found that more followers of fast-food brands used unhealthy hashtags than healthy ones on Instagram, and more followers of low-calorie drink brands used healthy hashtags than unhealthy ones on Twitter. The link between engagement with digital food and beverage advertising and obesity rates or hashtag usage is concerning given the rise of the sector’s advertising on social media [14].

Our findings extend previous research on social-media-based food advertising. One study identified a positive correlation between youth-targeted advertising practices and the number of adolescent followers of those 27 brands on Twitter [15]. Other studies have found that corporations are utilizing social media platforms such as Instagram, to convert individual social media users into positive advertisers for their unhealthy products [19]. Adolescents have become primary drivers for said products, and their tendency to interact with brands in ways that mimic interactions between friends makes youth-targeted fast-food and sugary beverage brand social media ads much more harmful [18]. These results contribute to the literature because we identified a positive correlation between state-level obesity rates and the number of followers of food and beverage brands.

Our research also differs from previous work in important ways. Previous research on how adolescents engage with food and drink brands examined social media posts themselves [17,18,19,31] and used self-reported engagement data. In this study, however, we correlated engagement with obesity rates using purchased, more objective engagement data instead of self-reported data [16,17,18].

This work shows that the digital food environment, similar to the physical food environment, is associated with obesity rates. Physical food environments, characterized in part by the greater availability and advertising of unhealthy foods, are thought to increase rates of obesity by creating a “toxic food environment.” [32,33]. For example, a higher fast-food restaurant density is associated with greater obesity rates, and a higher supermarket density is associated with lower obesity rates [34,35]. Similarly, the presence of physical food and drink advertisements is associated with greater rates of obesity within a community [36]. On an individual level, both physical and digital food cues can contribute to weight gain across experimental and prospective studies [37,38]. As such, digital and physical food environments may be causally related to obesity.

This study has some limitations and several strengths. Although we classified each of the 27 included brands into one of three categories based on their major products, some brands have sub-brands featuring lower calorie items that are not treated separately in the data—excluding Coca-Cola. Therefore, it is possible that a brand’s followers include more health-minded individuals who engage only with sub-brands. It is also possible that “healthy” hashtags were overcounted, given that all sports hashtags were coded as “healthy”. Some users may have employed sports-related content in their posts for non-self-related content, i.e., attending sports events, which is difficult to distinguish within the dataset. Another limitation is that a series of ANCOVAs were conducted with 26 brands that are classified as low-calorie, sugary beverage, or fast-food brands. This resulted in each category having a relatively smaller sample size of brands for statistical analysis. Nevertheless, our results captured the overview of the relationship between consumers’ engagement with the brands’ social media accounts and their usage of hashtags. Finally, there is an inherent limitation in acquiring demographic data from Demographics Pro. Considering the results are inferences based on consumers’ networks, consumption, and language, they may not accurately reflect the actual demographics of users. By including data from a company that uses proprietary algorithms, we are limited in our ability to achieve a complete replicability of methods and detect errors. Despite this, we feel comfortable using data from Demographics Pro because capturing these data would be too time-intensive and cost-prohibitive otherwise, they filter out bots (i.e., artificial social media followers that brands can use to inflate their follower counts), they are used by a wide range of well-known brands and companies, and their methods have been published in other academic research [39,40,41].

## 5. Conclusions

Our findings suggest that following unhealthy food and beverage brands on social media correlates with increased obesity rates throughout the United States. This correlation does not imply causation. Rather, it suggests that more experimental research should be conducted on the digital food environment and its population-level effects. The exponential increase in social-media-based food advertising in the past decade, ref. [14], coupled with growing consumer engagement on fast-food and sugary drink brands’ social media accounts, ref. [15], is particularly concerning given that exposure to advertising is associated with an increased consumption of fast food, sugary drinks, and low-calorie beverages among youth [41]. Despite this increasing public health concern, studies have shown that there is a lack of comprehensive policies in place to limit unhealthy food advertisement on all major social media platforms [42]. Our study helps solidify the need for holistic regulations that limit the marketing of unhealthy foods across the digital food environment.

## Figures and Tables

**Table 1:** The mean number of followers of popular food and beverage brands (*n* = 26) by state in the U.S.

	Instagram							Twitter							Obesity Rates
	Low-Calorie Drink Brands (*n* = 4)		Sugary Drink Brands (*n* = 9)		Fast-Food Brands (*n* = 10)		Any	Low-Calorie Drink Brands (*n* = 4)		Sugary Drink Brands (*n* = 7)		Fast-Food Brands (*n* = 6)		Any	
	*n*	%	*n*	%	*n*	%	%	*n*	%	*n*	%	*n*	%	%	%
Mississippi	298	0.95	6585	0.64	10,228	0.88	0.41	1260	0.97	2890	0.77	13,324	0.97	0.41	39.5
West Virginia	138	0.48	4694	0.41	7449	0.51	0.18	841	0.70	2978	0.67	9257	0.72	0.16	39.5
Arkansas	322	0.77	7977	0.78	13,386	1.02	0.44	1073	0.73	3079	0.76	12,304	0.97	0.40	37.1
Louisiana	539	1.30	11,163	1.28	18,037	1.43	0.88	1296	1.70	4406	1.13	17,158	1.28	0.68	36.8
Kentucky	412	1.40	11,748	1.16	18,687	1.41	0.59	1725	1.58	6199	1.46	21,934	1.72	0.58	36.6
Alabama	628	2.05	15,414	1.66	27,007	2.00	1.20	2907	2.30	7022	1.70	25,579	2.00	1.37	36.2
Iowa	207	0.68	8291	0.72	13,247	0.69	0.37	997	0.83	4993	1.04	13,337	1.03	0.35	35.3
North Dakota	85	0.17	1718	0.14	2575	0.13	0.04	213	0.28	994	0.21	2192	0.20	0.03	35.1
Missouri	554	1.63	18,582	1.80	32,173	2.18	1.30	2234	2.28	9445	2.17	29,021	2.38	1.33	35.0
Oklahoma	298	0.80	11,763	1.13	18,191	1.14	0.67	979	0.98	4980	1.10	15,054	1.22	0.59	34.8
Texas	2464	7.50	94,086	9.23	145,352	9.34	7.40	7502	7.65	34,994	7.96	124,858	10.15	6.52	34.8
Kansas	180	0.65	7542	0.69	11,758	0.72	0.37	824	0.73	4072	0.87	10,973	0.87	0.40	34.4
Tennessee	769	2.15	22,320	2.09	38,069	2.48	1.35	2861	2.75	9445	2.30	36,334	2.87	1.35	34.4
South Carolina	589	1.78	15,936	1.54	24,847	1.70	1.04	1795	1.55	6856	1.61	22,814	1.78	0.95	34.3
Indiana	613	1.45	18,593	1.70	33,068	2.13	1.26	2727	2.28	10,097	2.34	30,941	2.50	1.37	34.1
Nebraska	205	0.47	5808	0.48	6503	0.41	0.28	638	1.18	3095	0.64	7791	0.62	0.34	34.1
Ohio	1192	3.38	40,711	3.88	64,599	4.46	2.57	4838	4.50	21,615	4.89	66,161	5.17	2.55	34.0
DC	444	1.25	7298	0.83	13,143	0.83	0.74	1023	1.13	2942	0.74	9956	0.83	1.07	33.5
Michigan	863	2.50	29,865	2.68	40,186	2.67	1.95	3226	2.80	15,104	3.37	41,043	3.17	2.09	33.0
North Carolina	863	2.68	28,501	2.72	41,768	2.78	1.79	3135	2.83	12,268	2.93	39,496	3.18	1.66	33.0
Georgia	2033	8.80	36,895	4.11	64,622	4.51	2.63	4617	5.03	14,229	3.59	53,018	4.33	2.60	32.5
New Mexico	112	0.27	4299	0.33	4715	0.29	0.28	252	0.38	1763	0.39	4138	0.33	0.22	32.3
Wisconsin	450	1.40	17,309	1.70	25,047	1.54	0.83	1920	1.53	8440	1.86	18,824	1.57	0.94	32.0
Illinois	1318	3.90	37,753	3.63	62,506	3.97	3.17	4395	4.18	19,107	4.59	52,640	4.20	3.32	31.8
Maryland	419	1.18	13,757	1.33	21,491	1.51	1.12	1377	1.20	5861	1.43	18,954	1.55	1.04	30.9
Pennsylvania	1305	3.55	41,349	3.84	62,667	4.34	3.11	4141	4.33	19,969	4.60	56,490	4.58	2.80	30.9
Florida	1968	6.58	73,767	6.40	107,993	6.89	6.74	6091	5.58	24,176	5.56	74,199	6.00	5.72	30.7
Maine	59	0.20	3193	0.28	4618	0.25	0.24	543	0.40	1853	0.39	4034	0.35	0.31	30.4
Virginia	670	1.78	22,200	2.20	37,890	2.38	2.04	2151	2.58	9834	2.23	31,300	2.48	1.87	30.4
Minnesota	534	1.48	19,059	1.41	21,664	1.29	0.88	1765	1.88	8706	1.74	19,481	1.72	0.95	30.1
South Dakota	69	0.17	1968	0.14	2804	0.15	0.06	351	0.27	1088	0.23	2310	0.22	0.04	30.1
Oregon	227	0.70	14,009	1.01	14,626	0.84	0.80	1194	0.83	4280	0.97	9399	0.80	0.68	29.9
New Hampshire	87	0.20	3313	0.27	2838	0.19	0.11	446	0.33	1415	0.27	3188	0.27	0.10	29.6
Alaska	42	0.10	2257	0.14	1493	0.08	0.08	188	0.17	763	0.16	1485	0.13	0.10	29.5
Arizona	501	1.38	23,629	1.87	25,303	1.48	1.44	1870	1.50	7928	1.66	18,401	1.53	1.33	29.5
Nevada	279	0.83	13,736	1.06	14,534	0.92	1.05	1175	0.87	5859	1.16	11,583	0.93	1.09	29.5
Wyoming	14	0.10	1015	0.09	1239	0.12	0.01	78	0.23	722	0.14	1005	0.08	0.00	29.0
Washington	386	1.13	24,409	1.79	34,609	1.56	1.56	1801	1.33	7317	1.54	17,475	1.45	1.40	28.7
Idaho	71	0.23	4015	0.24	3095	0.19	0.14	353	0.30	1541	0.30	2963	0.23	0.19	28.4
Utah	747	1.57	19,438	1.20	9033	0.66	0.80	1002	0.80	4713	0.89	8375	0.63	0.65	27.8
Rhode Island	131	0.38	3880	0.34	5801	0.39	0.29	526	0.43	1572	0.37	4578	0.35	0.19	27.7
New York State	3441	11.08	71,614	7.01	102,088	6.61	8.00	7821	9.05	30,662	7.27	78,280	6.13	8.53	27.6
Vermont	41	0.15	1910	0.14	1355	0.06	0.03	122	0.07	924	0.14	1056	0.08	0.00	27.5
Connecticut	330	0.83	14,120	1.21	19,412	1.26	0.96	1105	1.08	4291	1.04	11,328	0.93	0.71	27.4
Montana	54	0.20	4381	0.28	2727	0.21	0.17	227	0.17	1484	0.29	2413	0.18	0.17	26.9
California	4171	12.83	202,417	15.73	235,967	13.09	18.49	11,169	12.48	59,213	12.04	122,183	9.85	17.50	25.8
Massachusetts	736	2.20	22,101	1.81	29,916	1.85	2.00	2253	2.38	10,186	2.19	23,520	1.82	1.79	25.7
New Jersey	770	2.35	27,580	2.43	36,144	2.47	2.23	2129	2.23	8954	2.14	24,926	1.90	1.72	25.7
Hawaii	103	0.33	5265	0.39	6051	0.39	0.35	312	0.23	1405	0.26	3034	0.22	0.31	24.9
Delaware	75	0.17	2476	0.22	3088	0.22	0.14	257	0.20	873	0.20	2744	0.20	0.10	24.7
Colorado	363	1.18	29,740	1.79	23,108	1.30	1.32	1093	1.25	9067	1.69	16,405	1.32	1.34	23.0

Note: Any = Users who followed any account on Instagram or Twitter.

**Table 2:** Comparison of the mean number of followers of popular food and beverage brands (*n* = 26) who used a healthy or unhealthy hashtag.

	Instagram					Twitter				
	Total Followers	Followers Who Used a Healthy Hashtag (*n* = 79)		Followers Who Used an Unhealthy Hashtag (*n* = 51)		Total Followers	Followers Who Used a Healthy Hashtag (*n* = 57)		Followers Who Used an Unhealthy Hashtag (*n* = 11)	
	*n*	*n*	%	*n*	%	*n*	*n*	%	*n*	%
**Low-Calorie Drink Brands**										
Coca Cola Life	6049	58	0.96	70	1.16	-	-	-	-	-
Coke Zero	98,742	457	0.46	507	0.51	253,914	404	0.16	149	0.07
Dasani Water	-	-	-	-	-	14,327	125	0.87	11	0.07
Diet Coke	79,181	658	0.84	808	1.03	305,944	428	0.15	197	0.05
Smart Water	48,841	672	1.37	531	1.10	5493	30	0.56	7	0.13
Subtotal	232,813	1845	-	1916	-	579,678	988	-	364	-
**Sugary Drink Brands**										
Coca Cola	2,592,532	19,340	0.74	22,667	0.87	-	-	-	-	-
Dr. Pepper	536,521	2816	0.52	3473	0.65	-	-	-	-	-
Fanta	517,501	1746	0.34	2074	0.40	157,722	178	0.11	101	0.06
Gatorade	1,167,065	8716	0.75	4872	0.41	331,396	772	0.23	250	0.07
Monster Energy	5,027,096	55,012	1.09	41,896	0.83	3,198,430	4168	0.13	2337	0.07
Mountain Dew	425,378	2948	0.69	2618	0.62	564,512	805	0.15	466	0.06
Pepsi	1,438,122	7758	0.54	8788	0.61	-	-	-	-	-
Red Bull	10,293,957	138,618	1.35	103,051	1.01	2,101,969	3326	0.16	1485	0.05
Sprite	869,636	3436	0.40	3613	0.41	284,233	376	0.13	224	0.08
Vitamin Water	-	-	-	-	-	161,000	331	0.21	159	0.10
Subtotal	2,2867,808	240,389	-	193,052	-	6,799,262	9955	-	5022	-
**Fast-Food Brands**										
Burger King	1,623,786	10,981	0.68	15,235	0.94	1,713,262	2062	0.12	1422	0.07
Chick-fil-A	1,256,639	10,272	0.82	10,829	0.86	958,494	1362	0.14	706	0.08
Dairy Queen	473,337	2857	0.61	3664	0.78	477,430	788	0.16	347	0.07
Denny’s Diner	-	-	-	-	-	520,034	629	0.11	396	0.08
KFC	1,357,038	7206	0.53	9632	0.71	-	-	-	-	-
McDonald’s	3,342,259	20,098	0.61	26,514	0.79	-	-	-	-	-
Pizza Hut	1,527,842	9894	0.65	13,577	0.89	-	-	-	-	-
Starbucks	17,425,064	198,844	1.14	229,017	1.32	-	-	-	-	-
Subway	1,030,818	5022	0.48	5992	0.58	2,361,855	3129	0.14	1838	0.08
Taco Bell	1,274,017	9241	0.73	11,234	0.88	-	-	-	-	-
Wendy’s	834,654	4424	0.53	5800	0.70	3,000,678	3530	0.12	2273	0.07
Subtotal	30,145,454	278,839	-	331,493	-	9,031,753	11,500	-	6982	-
**Any**	**-**	-	0.58	-	0.55	-	-	0.09	-	0.04

Note: Any = Users who followed any account on Instagram or Twitter.

## Data Availability

The data presented in this study are available on request from the corresponding author. The data are not publicly available due to accessibility rules set by Demographics Pro.

## Supplementary Materials

The following are available online at https://www.mdpi.com/article/10.3390/ijerph182312785/s1, Table S1: Results of regression analysis for relationship between the number of followers of popular and beverage brands (*n* = 26) and obesity rates by state in the USA, Table S2: Results of ANCOVA for differences in the mean number of followers among hashtags (healthy or unhealthy) or brands (low-calorie drink, sugary drink, or fast food), Table S3: Results of simple main effect analysis for hashtags, Table S4: Results of simple main effect analysis for brands, Table S5: Results of pairwise comparison between hashtags (healthy and unhealthy) at each brand, Table S6: Results of pairwise comparison between brands (low-calorie drink, sugary drink, and fast food) at each hashtag.
