# Supplementary material for: Associations between State-Level Obesity Rates, Engagement with Food Brands on Social Media, and Hashtag Usage
Source: Int J Environ Res Public Health. 2021 Dec 3;18(23):12785. doi: 10.3390/ijerph182312785 (PMC8657578; doi:10.3390/ijerph182312785)
Supplement: Supplementary file 1 [file ijerph-18-12785-s001.zip › ijerph-1420146-supplementary.pdf]

Table S1. Results of regression analysis for relationship between the number of followers of popular and beverage brands (n = 26) and obesity rates by state in USA

	Predictors	Estimate	*SE*	*p*	*F*	R^2
Instagram	(Intercept)	31.397 ***	0.671	< .001	*F* (2, 48) = 1.679	0.065
	Any	- 0.721	0.422	.09		
	Low-Calorie Drink	0.572	0.458	.22		
	(Intercept)	30.507 ***	0.745	< .001	*F* (2, 48) = 4.524	0.159
	Any	- 2.583 **	0.897	.006		
	Sugary Drink	2.625 *	0.988	.01		
	(Intercept)	30.431 ***	0.696	< .001	*F* (2, 48) = 6.310	0.208
	Any	- 1.894 ***	0.536	< .001		
	Fast Food	2.073 **	0.640	.002		
Twitter	(Intercept)	30.563 ***	0.723	< .001	*F* (2, 48) = 4.653	0.162
	Any	- 1.680 **	0.556	.004		
	Low-Calorie Drink	1.753 **	0.644	.009		
	(Intercept)	30.475 ***	0.777	< .001	*F* (2, 48) = 3.983	0.142
	Any	- 1.553 **	0.558	.008		
	Sugary Drink	1.694 *	0.686	.02		
	(Intercept)	30.521 ***	0.693	< .001	*F* (2, 48) = 5.766	0.194
	Any	- 1.219 **	0.361	.001		
	Fast Food	1.396 **	0.452	.003		

Note. Any = Users who followed any account on Instagram or Twitter. *SE* = Standard Error. *** $p < .001$, ** $p < .01$, * $p < .05$ for two-tailed tests.

Table S2: Results of ANCOVA for differences in the mean number of followers among hashtags (healthy or unhealthy) or brands (low calorie drink, sugary drink, or fast food).

Instagram					Twitter				
Factor		*F*	*p*	η_G^2	Factor		*F*	*p*	η_G^2
Any	***	F (1, 383) = 2334.538	< .001	0.859	Any	***	F (1, 197) = 225.195	< .001	0.533
Hashtags	**	F (1, 383) = 9.379	.002	0.024	Hashtag		F (1, 197) = 2.198	.14	0.011
Brands	***	F (2, 383) = 33.537	< .001	0.149	Brand	***	F (2, 197) = 25.916	< .001	0.208
Hashtags x Brands	***	F (2, 383) = 7.383	< .001	0.037	Hashtag x Brand	*	F (2, 197) = 4.740	.01	0.046

Note. Any = Users who followed any account on Instagram or Twitter. *** $p < .001$, ** $p < .01$, * $p < .05$ for two-tailed tests.

Table S3: Results of simple main effect analysis for hashtags

	Instagram					Twitter				
Brand	Factor		F	p	η_G^2	Factor		F	p	η_G^2
Low-Calorie Drink	Any	***	F (1, 127) = 1352	< .001	0.914	Any	***	F (1, 65) = 182	< .001	0.737
	Hashtags		F (1, 127) = 3.83	.16	0.029	Hashtags		F (1, 65) = 3.46	.20	0.050
Sugary Drink	Any	***	F (1, 127) = 458	< .001	0.783	Any	***	F (1, 65) = 214	< .001	0.767
	Hashtags		F (1, 127) = 0.853	1.00	0.007	Hashtags		F (1, 65) = 0.668	1.00	0.010
Fast Food	Any	***	F (1, 127) = 1168	< .001	0.902	Any	***	F (1, 65) = 97.4	< .001	0.600
	Hashtags	***	F (1, 127) = 32.2	< .001	0.202	Hashtags		F (1, 65) = 0.012	1.00	0.000177

Note. Any = Users who followed any account on Instagram or Twitter. *** $p < .001$ for two-tailed tests. All p-values are corrected with the Bonferroni method.

Table S4: Results of simple main effect analysis for brands

	Instagram					Twitter				
Hashtag	Factor		F	p	η_G^2	Factor		F	p	η_G^2
Healthy	Any	***	F (1, 233) = 891	< .001	0.793	Any	***	F (1, 167) = 191	< .001	0.534
	Brands	***	F (2, 233) = 17.2	< .001	0.129	Brands	***	F (2, 167) = 26.2	< .001	0.238
Unhealthy	Any	***	F (1, 149) = 2074	< .001	0.933	Any	***	F (1, 29) = 22.5	< .001	0.437
	Brands	***	F (2, 149) = 32.9	< .001	0.307	Brands		F (2, 29) = 0.113	1.00	0.008

Note. Any = Users who followed any account on Instagram or Twitter. *** $p < .001$ for two-tailed tests. All p-values are corrected with the Bonferroni method.

Table S5: Results of pairwise comparison between hashtags (healthy and unhealthy) at each brand

	Instagram			Twitter		
Brand	Estimate	*SE*	*p*	Estimate	*SE*	*p*
Low-Calorie Drink	- 0.0714	0.0438	.10	0.252 ***	0.0751	< .001
Sugary Drink	0.0384	0.0438	.38	- 0.0126	0.0751	.87
Fast Food	- 0.199 ***	0.0438	< .001	- 0.0450	0.0751	.55

Note. The results on grey background are computed for significant simple main effect of Hashtag. Estimate = Estimated difference between the two estimated marginal means. *SE* = Standard Error. *** $p < .001$ for two-tailed tests. All *p*-values are corrected with the Bonferroni method.

Table S6: Results of pairwise comparison between brands (low calorie drink, sugary drink, and fast food) at each hashtag

		Instagram			Twitter		
Hashtag	Brand Comparison	Estimate	*SE*	*p*	Estimate	*SE*	*p*
Healthy	Low-Calorie Drink vs Sugary Drink	0.193 ***	0.0388	< .001	0.274 ***	0.0426	< .001
	Low-Calorie Drink vs Fast Food	0.231 ***	0.0388	< .001	0.302 ***	0.0426	< .001
	Sugary Drink vs Fast Food	0.0378	0.0388	.99	0.0279	0.0426	1.00
Unhealthy	Low-Calorie Drink vs Sugary Drink	0.303 ***	0.0482	< .001	0.00909	0.0969	1.00
	Low-Calorie Drink vs Fast Food	0.104	0.0482	.10	0.00455	0.0969	1.00
	Sugary Drink vs Fast Food	- 0.200 ***	0.0482	< .001	- 0.00455	0.0969	1.00

Note. The results on grey background are computed for significant simple main effect of brands. Estimate = Estimated difference between the two estimated marginal means. *SE* = Standard Error. *** $p < .001$ for two-tailed tests. All *p*-values are corrected with the Bonferroni method.
